# Regulatory T cells promote glioma cell stemness through TGF-β–NF-κB–IL6–STAT3 signaling

**DOI:** 10.1007/s00262-021-02872-0

**Published:** 2021-02-12

**Authors:** Shasha Liu, Chaoqi Zhang, Boqiao Wang, Huanyu Zhang, Guohui Qin, Congcong Li, Ling Cao, Qun Gao, Yu Ping, Kai Zhang, Jingyao Lian, Qitai Zhao, Dan Wang, Zhen Zhang, Xuan Zhao, Li Yang, Lan Huang, Bo Yang, Yi Zhang

**Affiliations:** 1 Biotherapy Center, The First Affiliated Hospital of Zhengzhou University, 1 Jianshe East Road, Zhengzhou, 450052 Henan China; 2 Cancer Center, The First Affiliated Hospital of Zhengzhou University, Zhengzhou, 450052 Henan China; 3 Cancer/Cancer Hospital, Chinese Academy of Medical Sciences and Peking Union Medical College, Beijing, 100021 China; 4 Henan University of Chinese Medicine, Zhengzhou, 450052 Henan China; 5 Department of Neurosurgery, The First Affiliated Hospital of Zhengzhou University, Zhengzhou, 450052 Henan China; 6 Henan Key Laboratory for Tumor Immunology and Biotherapy, Zhengzhou, 450052 Henan China; 7 School of Life Sciences, Zhengzhou University, Zhengzhou, 450052 Henan China

**Keywords:** Glioma, Tregs, Glioma stem cell, TGF-β, Tocilizumab

## Abstract

Glioma stem cells (GSCs) contribute to the malignant growth of glioma, but little is known about the interaction between GSCs and tumor microenvironment. Here, we found that intense infiltration of regulatory T cells (Tregs) facilitated the qualities of GSCs through TGF-β secretion that helped coordinately tumor growth. Mechanistic investigations indicated that TGF-β acted on cancer cells to induce the core cancer stem cell-related genes CD133, SOX2, NESTIN, MUSASHI1 and ALDH1A expression and spheres formation via NF-κB–IL6–STAT3 signaling pathway, resulting in the increased cancer stemness and tumorigenic potential. Furthermore, Tregs promoted glioma tumor growth, and this effect could be abrogated with blockade of IL6 receptor by tocilizumab which also demonstrated certain level of therapeutic efficacy in xenograft model. Additionally, expression levels of CD133, IL6 and TGF-β were found to serve as prognosis markers of glioma patients. Collectively, our findings reveal a new immune-associated mechanism underlying Tregs-induced GSCs. Moreover, efforts to target this network may be an effective strategy for treating glioma.

**Supplementary Information:**

The online version of this article (10.1007/s00262-021-02872-0) contains supplementary material, which is available to authorized users.

## Introduction

Glioma is the most common primary brain tumor in humans, characterized by a high invasion rate that results in diffuse tumor infiltration throughout the central nervous system (CNS) [1]. Even when multiple treatment approaches are used, including surgery and rigorous chemotherapy and radiation therapy, the median overall survival rate is only 15–19 months, and the survival rate is > 5%, because of the high tumor recurrence rate [2–5]. Growing evidence suggests that glioma stem cells (GSCs) are responsible for glioma initiation, recurrence, and chemo- and radio-resistance [6, 7]. Thus, there is considerable value in exploring the mechanisms that contribute to glioma development and therapy resistance, particularly those related to the production of GSCs. Therapies to target GSCs have been mainly focused on the elimination of GSCs, by targeting surface markers and GSC stemness maintained by particular pathways.

Recently, interest has emerged regarding the crosstalk between the cancer stem cell (CSC) niche and other cellular components of the tumor microenvironment (TME), in particular immune cells [8, 9]. Various studies have found that cytokines produced by the infiltrated cells in the TME can have important effects on CSC stemness. For example, tumor-associated macrophages (TAMs) produced IL6 to promote expansion of CSCs via STAT3 signaling in human hepatocellular carcinoma [10]. Foxp3^+^IL-17^+^ T cells promoted development of CSCs in colorectal cancer through the activation of Akt and MAPK pathways [11]. Myeloid-derived suppressor cells (MDSCs) were able to promote stem-like qualities to breast cancer cells through IL6/STAT3 and NO/NOTCH crosstalk signaling [12]. IL-22^+^CD4^+^ T cells were able to promote colorectal cancer stemness via the STAT3-DOT1L pathway [13]. CD10^+^GPR77^+^ cancer-associated fibroblasts (CAFs) sustained cancer stemness and promoted tumor chemoresistance [14]. Nonetheless, the interactions between CSCs and regulatory T cells (Tregs), which play an important immunosuppressive role in the TME, are still poorly understood.

Tregs have become widely acknowledged as the central agent responsible for immunosuppression and tumor promotion in glioma [15]. They are elevated in the blood and tumors of glioma patients and animals with experimentally induced brain tumors [16]. Tumor infiltration by Tregs correlates with tumor grade, and in animal models, depletion of Tregs is associated with prolonged survival [17, 18]. So far, most evidence suggests that Tregs promote immune escape mainly via inhibition of effector T cells, by secreting various soluble factors such as IL-10 and TGF-β [19]. However, the mechanisms whereby Tregs alter the properties of GSCs in glioma are still obscure.

Our objective was to examine the interaction between Tregs and GSCs in patients with glioma, dissecting the underlying molecular mechanisms whereby Tregs promote GSCs via the TGF-β–NF-κB–IL6–STAT3 axis. We aimed to determine the therapeutic value of the IL6 receptor-blocking antibody, tocilizumab, a humanized anti-IL6 receptor drug approved by the FDA (USA) and SFDA (China), for targeting GSCs in glioma.

## Materials and methods

### Clinical subjects

In total, 86 patients with glioma were recruited, following approval by the Ethics Committee Board of the First Affiliated Hospital of Zhengzhou University, Zhengzhou, China. From these patients, 86 tumor samples and paired peripheral blood samples were collected during surgery at the First Affiliated Hospital of Zhengzhou University, between January 2013 and July 2016. In addition, 72 samples of adjacent normal noncancerous tissue were collected.

### Purification of CD25^+^CD4^+^ Treg cells

Human peripheral blood was obtained from glioma patients in accordance with local ethical committee approval. Peripheral blood mononuclear cells (PBMCs) were isolated by Ficoll density gradient (Tianjin HY, China) centrifugation, and CD4^+^ T cells were isolated by negative selection using the Human CD4^+^ T Cell Biotinylated Antibody Cocktail (R&D Systems). After isolation of CD4^+^ T cells, CD25 cells were purified using the anti-Human CD25 Biotinylated Antibody (R&D Systems). Starting with 10^8^ PBMCs, typically 2–3 × 10^6^ CD4^+^CD25^+^ Treg cells were isolated (90–95% purity).

### Cell culture

U251 and U87 cells (glioma cell lines) were purchased from the Chinese Academy of Sciences Cell Repertoire in Shanghai, China, and were maintained in high-glucose DMEM (HyClone, Logan, UT) supplemented with 10% fetal bovine serum (HyClone), 100 units/ml penicillin, and 100 μg/ml streptomycin, at 37 °C and 5% CO^2^ in a humidified incubator. For co-culture experiments, 24-well transwell chambers with 0.4-μm porous polycarbonate membrane (Costar, Corning, Inc.) were used, with 1 × 10^5^ U251 or U87 cells seeded in the lower chamber 1 d before co-culture, and 1 × 10^5^ Tregs added in the upper chamber. Humanized anti-IL6 receptor-blocking antibody (tocilizumab, Genentech, 5 μg/ml) was added in the indicated experiments to specific wells. For cell–cell contact assay, 1 × 10^5^ U251 cells and 1 × 10^5^ Tregs were co-cultured in regular 24-well plates without transwell chambers. After 24 h, tumor cells and Tregs were collected separately for analysis.

### Sphere-forming assay

The sphere-forming assay was performed as described previously [20]. Cells were cultured in serum-free DMEM/F12 medium (Invitrogen, USA) supplemented with 4 μg/ml heparin (Sigma, USA), 2% B27 supplement (1:50 dilution; GIBCO, USA), 20 ng/ml human recombinant epidermal growth factor (PeproTech, USA), 20 ng/ml human recombinant basic fibroblast growth factor (bFGF) (PeproTech, USA), 100 IU/ml penicillin and 100 μg/ml streptomycin, in an Ultra Low Attachment Culture Flask (Corning, USA). After culturing for 1 week, the number of spheres was counted under a microscope (Leica, Germany).

### Multiple-cytokine assay

The relative quantities of 13 different cytokines in the supernatant from the U251/Tregs co-cultured system and TGF-β-treated U251 glioma cells were analyzed using the LEGENDplex Human Th Cytokine Panel Kit (Biolegend, USA), following the manufacturer’s instructions. Supernatants (25 μl) from the U251/Tregs cocultured system, TGF-β-treated U251 glioma cells, and the 25 μl diluted standard sample, were co-cultured with the mixture containing detection antibodies, mixed capture beads, and assay buffer at room temperature on a shaker. After 2 h, 25 μl SA-PE was added to each tube and incubated on a shaker for another 30 min at room temperature. Mixed beads were then spun down, washed once with wash buffer, and re-suspended with 200 μl wash buffer. Samples and standards were read individually using flow cytometry. Relative amounts of the 13 cytokines in supernatants were analyzed according to the standard using the LEGENDplex Data Analysis Software.

### RNA extraction and quantitative real-time PCR

Total RNA from the U251/Tregs cultures and glioma patient tissues was extracted using Invitrogen TRIzol Reagent (Thermo Scientific, USA), according to manufacturer’s instructions. The concentration and purity of RNA were detected using a NanoDrop 2000 spectrophotometer (Thermo Scientific). Independently, RNA from each sample was reverse-transcribed using the PrimeScript RT reagent Kit (Takara, Japan). Subsequently, quantitative real-time PCR (RT-PCR) was performed using SYBR Premix Ex Taq II (Takara, Japan) in the StepOnePlus system (Applied Biosystems). Each experiment was performed in triplicate. Glyceraldehyde-3-phosphate dehydrogenase (GAPDH) was used as an endogenous control for normalization.

### Flow cytometry

U251 and U87 were suspended in flow buffer (PBS containing 2% fetal bovine serum) and incubated with anti-CD133 (BioLegend, San Diego, CA) against surface antigens for 30 min at 4 °C in the dark. Samples were then rinsed twice in flow buffer and analyzed using a BD FACSCanto II Flow Cytometer (Becton Dickinson, San Jose, CA). To detect nonspecific signals, concentration- and isotype-matched nonspecific antibodies were used.

### ELISA

Cell culture supernatants were obtained from plates of treated cells. ELISAs were processed using the Human IL6 ELISA Kit (R&D Systems) and Human TGF-β1 ELISA Kit (BioLegend), according to the manufacturer’s instructions. Absorbance at 450 nm was measured using a microplate reader.

### Western blotting

U251 cells were used for western blotting analysis, as described previously [21]. Briefly, whole cell lysates were prepared using RIPA lysis buffer with protease inhibitor cocktail (Sigma-Aldrich, USA) and phosphatase inhibitor cocktail 2 (Sigma-Aldrich, USA). Protein concentration was measured using a bicinchoninic acid assay method kit (Biyuntian, Jiangsu, China). Equal amounts of protein were loaded in 10% SDS-PAGE and then transferred from the gel to a polyvinylidene fluoride membrane (Bio-Rad, Hercules, CA). Membranes were blocked in 5% nonfat milk and incubated with primary antibodies overnight at 4 °C, followed by treatment with secondary antibody for 2 h at 37 °C. Primary antibodies used for western blotting were as follows: rabbit anti-human phosphorylated STAT3 (1:1000; Cell Signaling Technology, USA), NF-κB (1:1000; Cell Signaling Technology), rabbit anti-human CD133 (1:2000; Proteintech), SOX2 (1:4000; Proteintech, USA), NESTIN (1:1000; Proteintech, USA), mouse anti-human IL6 (1:2000; Proteintech, USA), and mouse anti-human β-actin (1:5000; Cell Signaling Technology), and were imaged using enhanced chemiluminescence (Thermo Fisher, USA), according to the manufacturer’s protocols.

### Stable lentiviral knockdown of TGFBR2 and IL6 in U251 cells and cell sorting

Stable knockdown of TGFBR2 and IL6 in U251 cells by short hairpin (sh) RNA was achieved using the pGV248-hu6-GFP^+^ Puro vector plasmid (Gene Pharma, Shanghai, China). Lentivirus production was conducted by transfection of HEK-293 T cells with pGV248-hu6-GFP-IL6RNAi, along with the packaging and envelope plasmids psPAX2 and pMD2.G, using Lipofectamine 3000 (Life Technologies), according to the manufacturer’s instructions. Virus particles were harvested 48 h after transfection. U251 cells were infected with lentivirus-transducing units using Polybrene (10 mg/ml) (Sigma). Expression of TGFBR2 and IL6 was confirmed by real-time RT-PCR and western blotting. Inserted sequences were confirmed by DNA sequencing. Transfected cells were sorted by flow cytometry using the MoFlo XDP cell sorter (Beckman Coulter, Brea, CA), based on the expression of green fluorescent protein.

### Immunohistochemistry

Paraffin-embedded glioma tissues and their paired adjacent noncancerous tissues were examined for CD133 expression (1:200; Abcam, Cambridge, MA). Sections were treated with 3% hydrogen peroxide (H_2_O_2_) and 5% bovine serum albumin and incubated overnight with primary antibodies at 4 °C. After incubation with horseradish peroxidase-conjugated secondary antibody for 1 h at 37 °C, sections were washed and counterstained with hematoxylin and visualized under a microscope (Olympus, Tokyo, Japan).

### Immunofluorescence

Immunofluorescence staining was used to determine whether the TGF-β was derived mainly from Tregs. Briefly, the paraffin-embedded glioma tissues were stained with Foxp3 (1:100; Abcam), CD163 (1:500; Abcam) and TGF-β (1:200; Abcam) antibodies. Fluorescein isothiocyanate- and Cy3-conjugated secondary antibodies (1:500; BioLegend) were used to detect the primary antibodies. Stained cells were counterstained with 4′6-diamidino-2-phenylindole (1:1000; Roche, Basel, Switzerland) and analyzed using an IX71 inverted fluorescence microscope (model 1003; Olympus).

### Animal model

To generate a subcutaneous xenograft mouse model, 20 female BALB/c nude mice (Vital River Laboratory Animal Technology Co. Ltd, Beijing, China) aged 5 weeks were randomly divided into four groups (five mice per group). All groups received hypodermic injections of either U251 or the mixture of U251 and Tregs (1:1, 5 × 10^6^ cells in 100 μl phosphate-buffered saline), with or without tocilizumab (20 mg/kg IP every week). Mice were inspected every 4 d, and tumor growth was evaluated by measuring the length and width of the tumor mass with calipers. Tumor growth was monitored twice per week, and at the end of 2 weeks of treatment, mice were killed. The tumor volume was calculated by the formula (length × width^2^)/2. Mice samples were blinded during data measurement. All animal procedures were conducted in accordance with the Guide for the Care and Use of Laboratory Animals and were approved by the Institutional Animal Care and Use committee of the First Affiliated Hospital of Zhengzhou University.

### Statistical analysis

Results are reported as mean ± SEM. Cell experiments were performed in triplicate, and a minimum of three independent experiments were evaluated. Differences were assessed for statistical significance using GraphPad Prism. The statistical significance of differences between groups was determined using Student’s *t* test, and differences between groups were estimated by log-rank test. Survival curves were plotted using the Kaplan–Meier method. *P* values < 0.05 were considered to be statistically significant.

## Results

### High CD133 expression is associated with worse prognosis in patients with glioma

**Fig. 1:**
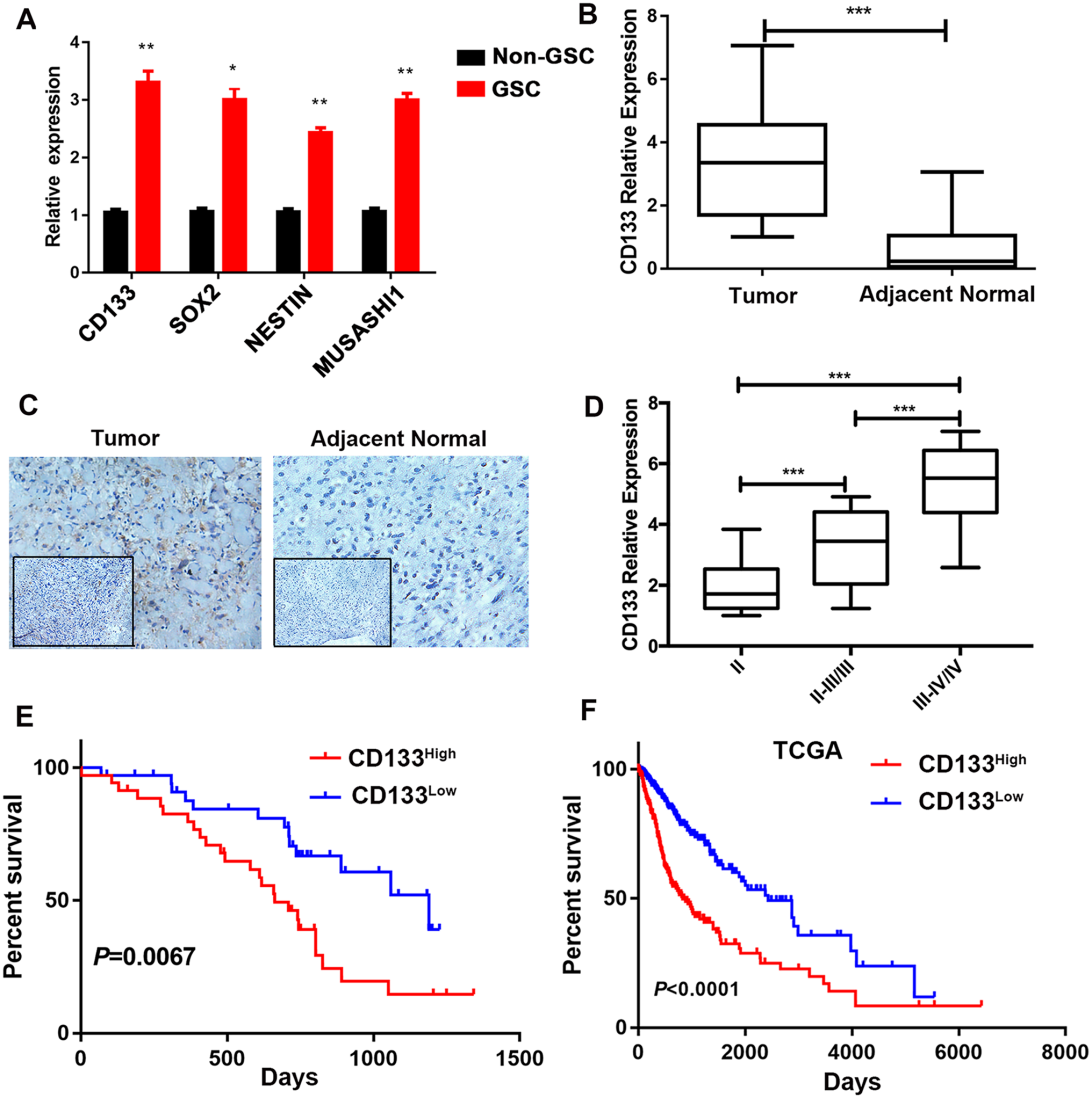
Expression of CD133 is enriched in glioma stem cells (GSCs) and indicates poorer survival in glioma patients.** a** Expression of *CD133*, *SOX2*, *NESTIN*, and *MUSASHI1*, analyzed using reverse-transcription PCR (RT-PCR) of the GSCs generated by the spheres of the glioma cells and non-GSCs (U251 cells).** b** Expression of CD133 was elevated in the tumor tissues (*n* = 72) compared to the adjacent normal tissues (*n* = 72). ** c**CD133 expression in tumor and adjacent tissues, detected using immunohistochemistry.** d** CD133 expression showing a positive correlation with WHO grade in glioma patients. We divided patients into ‘‘low’’ and ‘‘high’’ groups based on the median value of CD133. Kaplan–Meier of overall survival for patients with** e** low (*n* = 35) and high (*n* = 35) CD133 expression, and** f** low (*n* = 393) and high (*n* = 304) CD133 expression in the TCGA database. Results are based on three independent experiments. *P* < 0.05; **P < 0.01; ***P < 0.001

Studies have shown that CD133, the glioma stem cell marker, is elevated in glioma tissue and predicts poor prognosis in glioma patients [22–24]. Here, we first confirmed that CD133 expression is a marker of GSC. Previous studies have demonstrated that cancer stem cells can form spheres in vitro in a nonattached culture condition [25]. Therefore, we performed tumor sphere-formation assay using U251 cells and examined CD133 expression in tumor spheres and U251 cells. CD133 expression was enriched in GSCs. Similarly, expression of other GSC core markers such as SOX2, NESTIN and MUSASHI1 [8, 26] was higher in GSCs than in the non-GSCs (Fig. 1a). Second, the expression of CD133 was evaluated in glioma tumor tissues and adjacent normal tissues. CD133 expression was higher in tumor tissue than in adjacent normal tissues (Fig. 1b), validated by immunohistochemistry (Fig. 1c). To further interrogate the clinical significance of CD133 expression in glioma, we analyzed the association between CD133 expression and the survival of glioma patients. A positive association was found between CD133 expression and WHO tumor grade in glioma patients, whereas the expression of CD133 correlated negatively with prognosis (Fig. 1d, e). Data from The Cancer Genome Atlas (TCGA) database (http://cancergenome.nih.gov/) showed a similar result (Fig. 1f). Therefore, these findings demonstrate that the GSC marker CD133 plays an essential role in the prognosis of glioma patients.

### Tregs contribute to the expansion of GSC capacity

**Fig. 2:**
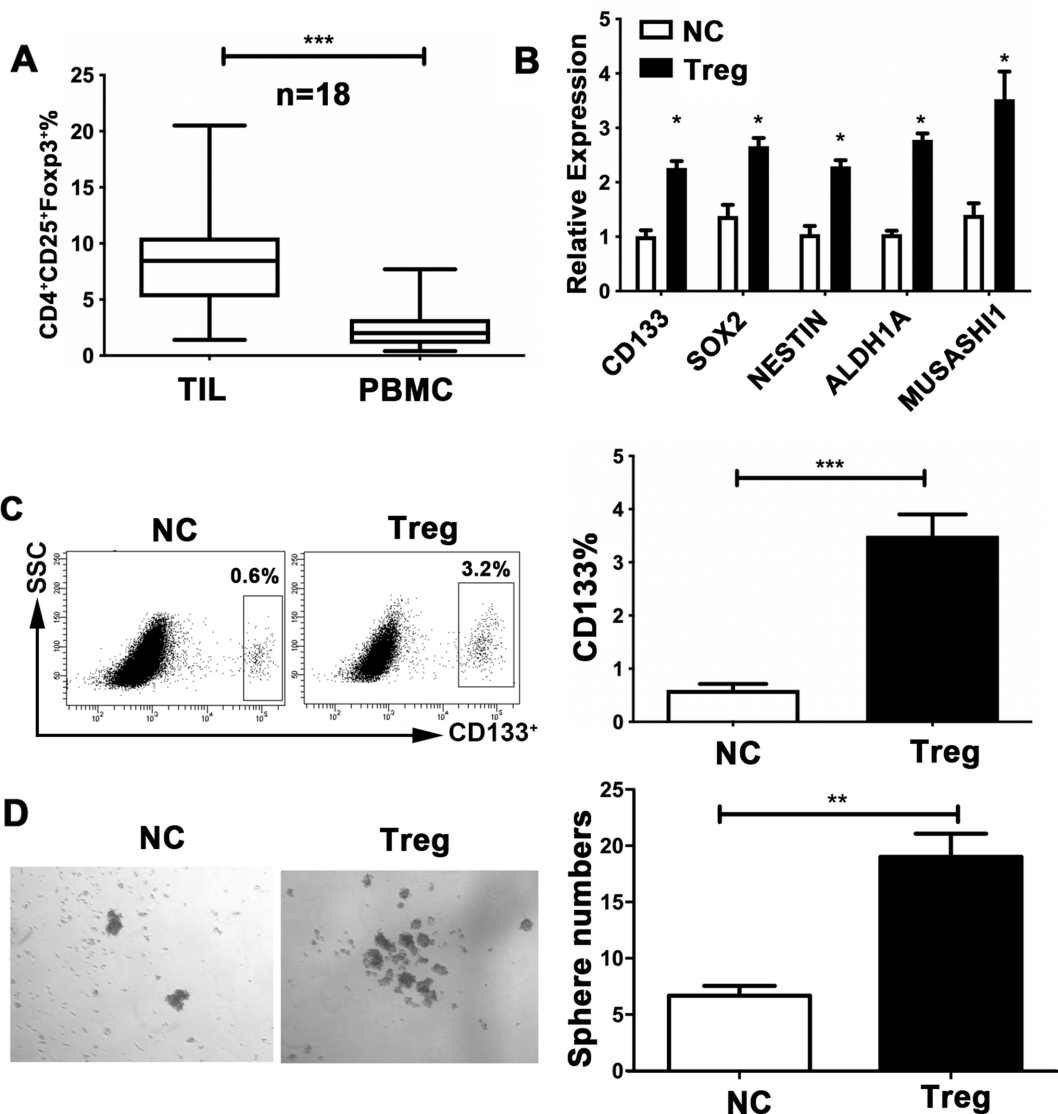
Regulatory T cells (Tregs) promote glioma stem cell (GSC) capacity in glioma cells.** a** Distribution of CD4^+^CD25^+^Foxp3^+^ Tregs in peripheral blood and tumor samples from glioma patients (*n* = 50), analyzed the percentage of total lymphocytes using flow cytometry. ** b***CD133*, *SOX2*, *NESTIN*, *ALDH1A*, and *MUSASHI1* expression in the U251 cells, detected using reverse-transcription PCR, following co-culture with Tregs (1:1; Transwell) sorted from the peripheral blood of the glioma patients.** c** CD133^+^ proportions of U251 cells, assessed by flow cytometry, following co-culture with Tregs for 24 h in transwell chambers.** d** U251 cells and Tregs co-cultured under sphere-forming conditions. The number of spheres per field was averaged from five randomly selected fields. Results are based on three independent experiments. *P < 0.05; **P < 0.01; ***P < 0.001.* NC* normal control

The immune system appears to play a crucial role in the control of cancer stemness [27, 28]. To gain insight into the cell types in the TME that are responsible for CD133 expression and GSC characteristics, we analyzed the relationships between CD133 expression and immunosuppressive cells, including MDSCs, TAMs, CAFs, and Tregs, in the glioma TME. Analysis of the TCGA database for glioma patients revealed a significantly positive association between CD133 and Foxp3, the specific marker for Tregs (Supplementary Fig. 1a, b, c, d). Consistently, we observed a positive correlation between CD133 and Foxp3 in our clinical glioma samples (Supplementary Fig. 1e). Additionally, we found a greater proportion of infiltrating CD4^+^CD25^+^Foxp3^+^ Tregs in the tumor tissue than in peripheral blood of glioma patients (Fig. 2a). Based on these findings, we suggest that Tregs might participate in establishing a supporting niche to sustain GSC expansion. Therefore, we sorted Tregs from the peripheral blood of glioma patients and cocultured them with the glioma cell-line U251. The function of Tregs was also validated by FACS (Supplementary Fig. 2a). The expression of CD133 in the U251 line was remarkably elevated in the cocultured system relative to cells cultured alone (Fig. 2b). The expression of multiple core GSC genes, including *CD133*, *SOX2*, *NESTIN*, *ALDH1A*, and *MUSASHI1*, was markedly elevated in cocultured U251 cells relative to those cultured alone (Fig. 2c). Following coculture, U251 cells formed significantly more spheres than the control (Fig. 2d). In line with this, the CD133^+^ subset of U87 cells was similarly expanded after cocultured with. Treg co-culture also increased the expression of GSC-related genes and resulted in a marked increase in the number of spheres formed in U87 cells (Supplementary Fig. 2b, c, d). Therefore, Treg abundance may contribute to the expansion of GSC capacity in glioma.

### Tregs Induce GSC Enrichment by Secreting TGF-β

**Fig. 3:**
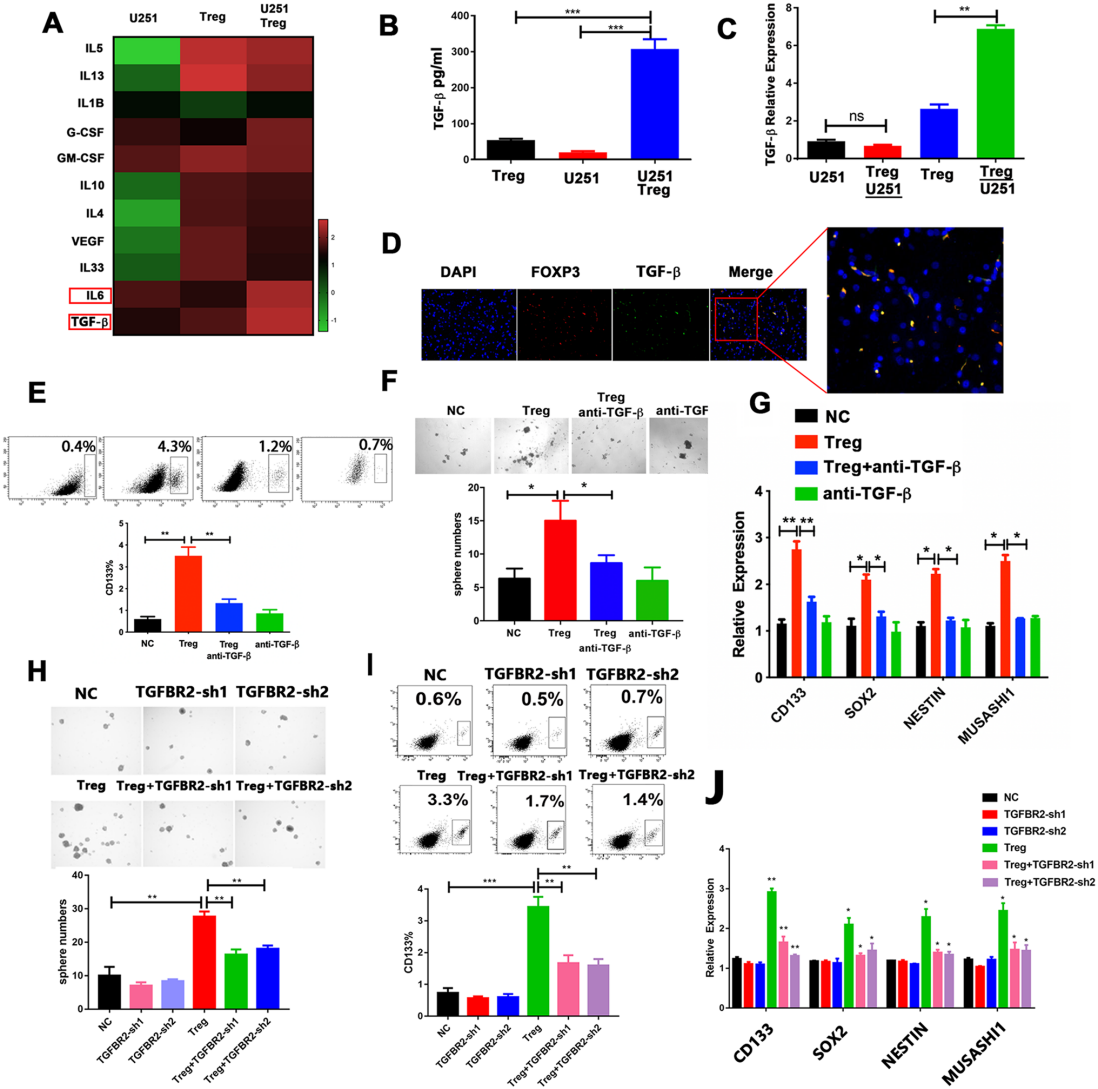
Regulatory T cells (Tregs) secrete TGF-β which promotes GSC expansion.** a** A multiple-cytokine kit was used to detect cytokines in the supernatants from the U251/Tregs co-culture and from the U251 and Tregs cultures alone.** b** The TGF-β protein level, validated using ELISA.** c** TGF-β expression in cells from the co-cultured system and from the U251 and Tregs cultures alone, based on reverse-transcription PCR (RT-PCR).** d** Immunofluorescence localization analysis of Foxp3 (red) and TGF-β (green) in glioma tissues; colocalization of Foxp3 and TGF-β is indicated in yellow. Scale bars: 200 μm.** e** CD133^+^ proportions in U251 cells co-cultured with Tregs in the presence of TGF-β-neutralizing antibody (1 μg/ml) and of control cells.** f** The number of spheres generated from U251 cells co-cultured with Tregs, with or without the TGF-β-neutralizing antibody (1 μg/ml). The number of spheres per field was averaged from five randomly selected fields.** g** Expression of cancer stemness genes *CD133*, *SOX2*, *NESTIN*, and *MUSASHI1* in U251 cells co-cultured with Tregs in the presence of TGF-β-neutralizing antibody (1 μg/ml) and of control cells, determined using RT-PCR.** h** Sphere numbers in U251 cells transfected with shTGFBR2 and control cells with or without Tregs. The number of spheres per field was averaged from five random fields.** i** CD133^+^ proportions in shTGFBR2-U251 cells and control cells with or without Tregs, analyzed using flow cytometry.** j** Expression of *CD133*, *SOX2*, *NESTIN*, and *MUSASHI1* in shTGFBR2-U251 cells and control cells with or without Tregs, evaluated using RT-PCR analysis. Results are based on three independent experiments. *P < 0.05; **P < 0.01; ***P < 0.001

To understand how Tregs induce GSC expansion, the supernatants from the U251 cell culture and the U251 and Tregs co-culture were analyzed. The most markedly elevated cytokines were TGF-β and IL6 (Fig. 3a). Strikingly similar results were observed in the protein level of TGF-β and IL6 (Fig. 3b and Supplementary Fig. 3a). To identify the source of TGF-β and IL6, RT-PCR was performed to detect expression of TGF-β and IL6 in U251 and Tregs obtained from co-cultured system separately and in the nonco-cultured U251 and Tregs cells. The expression of TGF-β was higher in Tregs obtained from the co-cultured system than in the other groups (Fig. 3c). In contrast, we found that IL6 expression was elevated only in U251 cells obtained from the co-cultured system (Supplementary Fig. 3b). Consistently, TGF-β was mainly derived from Tregs, not mainly from CD163^+^ TAMs in the glioma TME, based on immunofluorescence localization analysis (Fig. 3d and Supplementary Fig. 3c). Expression of CD133 in glioma patients was positively associated with TGF-β in glioma samples and in the TCGA database glioma samples (Supplementary Fig. 3d, e), suggesting that Tregs may augment GSC expansion by secreting TGF-β in glioma tissue.

To further address whether TGF-β is implicated in Treg-enhanced GSC function, a TGF-β receptor inhibitor was added to the Tregs and U251 co-culture assay. The increase in the proportion of CD133^+^ cells was attenuated by the TGF-β inhibitor (Fig. 3e). The TGF-β inhibitor also abrogated the elevated expression of the core cancer stemness genes and sphere formation induced by Tregs (Fig. 3f, g).

To further explore how TGF-β mediated Treg-induced GSC expansion, we examined the expression of three TGF-β receptors, TGFBR1, TGFBR2, and TGFBR3. The mRNA expression levels of these three receptors were measured using RT-PCR. TGFBR2 expression was much higher in U251 cells than in the other cells (Supplementary Fig. 3f). To determine the role of TGFBR2 in Treg-mediated GSC expansion, two pairs of TGFBR2 shRNAs were individually transfected into U251 cells to knock down endogenous TGFBR2 (Supplementary Fig. 3g). Consistently, silencing of TGFBR2 significantly abrogated Treg-induced GSC properties (Fig. 3h, i, j). To further address whether Tregs require cell–cell contact to induce GSC-promoted functions, Tregs were co-cultured with U251 via cell–cell contact. Glioma cells had elevated expression of core cancer stemness genes and an elevated proportion of CD133^+^ cells, after co-culture with Tregs (Fig. S4a, b). This elevated expression in the glioma cells generated significantly more spheres than were observed in the untreated cells (Fig. S4c). However, these effects also are attenuated by the TGF-β inhibitor (Fig. S4a, b, c). Therefore, these data suggest that Tregs contributed the GSCs properties in the manner of TGF-β secretion.

### TGF-β promotes GSC properties dependent on IL6-IL6R signaling in glioma

**Fig. 4:**
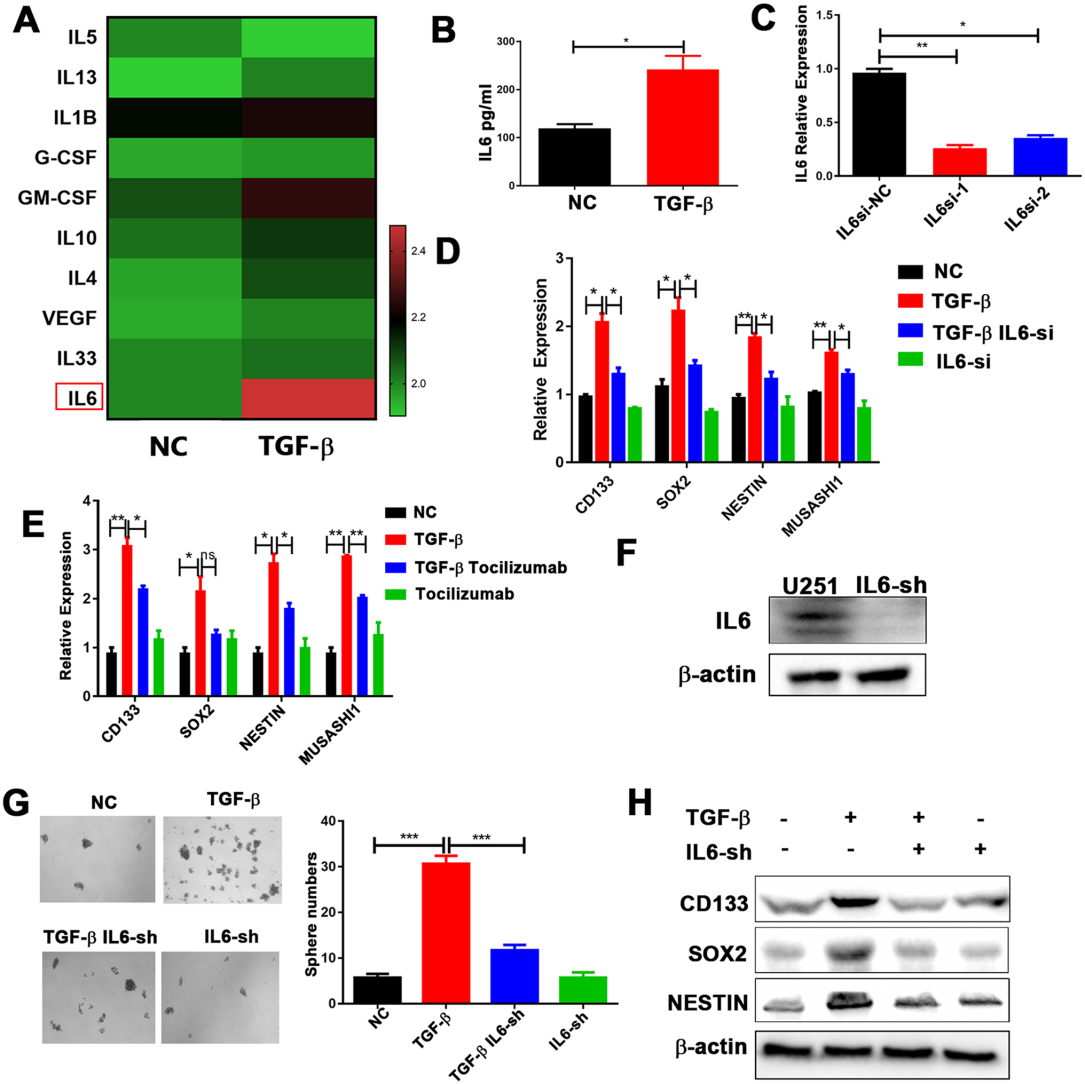
IL6-IL6R signaling mediates GSC properties induced by TGF-β.** a** Cytokine levels with or without TGF-β treatment (10 ng/ml), evaluated using a multiple-cytokine kit.** b** IL6 protein expression levels, validated using ELISA.** c** U251 cells were infected with siRNA to inhibit the expression of IL6. IL6 expression was examined by reverse-transcription PCR (RT-PCR). Expression of *CD133*, *SOX2*, *NESTIN*, and *MUSASHI1* in** d** U251 cells transfected with either si-scramble or siIL6, following TGF-β (10 ng/ml) treatment, and in** e** U251 cells treated with tocilizumab (5 μg/ml) following TGF-β treatment (10 ng/ml), evaluated using RT-PCR.** f** IL6 expression, examined by western blotting analysis, in U251 cells transfected with lentiviral sh-IL6.** g** Sphere-formation capacity of U251 cells transfected with either sh-scramble or sh-IL6 following TGF-β treatment (10 ng/ml), assessed by sphere-formation assay.** h** *CD133*, *SOX2*, and *NESTIN* expression in U251 cells transfected with either sh-scramble or shIL6 following TGF-β treatment (10 ng/ml), assessed using western blotting analysis. Results are based on three independent experiments. *P < 0.05; **P < 0.01; ***P < 0.001

To investigate the underlying mechanisms responsible for the effects of TGF-β on GSCs, we performed a multiple-cytokine assay to investigate cytokine secretion in the supernatants from U251 cells treated with TGF-β recombinant protein. Expression of IL6 was substantially elevated in the TGF-β treatment group, compared to the other groups (Fig. 4a). Similar elevation occurred at the protein level (Fig. 4b). The expression of TGF-β was positively correlated with IL6 expression in glioma samples (Supplementary Fig. 5a), further validated by analysis of the TCGA database (Supplementary Fig. 5b). In addition, as mentioned above, IL6 was mainly secreted by tumor cells in the co-culture system (Supplementary Fig. 3a, b); therefore, we hypothesized that IL6 would be involved in GSC self-renewal and would mediate the effects of TGF-β on GSCs. To further test this hypothesis, small interfering (si) IL6 was used to reduce IL6 expression (Fig. 4c). In the presence of TGF-β, the expression of core cancer stemness-associated genes was significantly reduced in U251 cells transfected with si-IL6 (Fig. 4d). Similar results were observed when tocilizumab was added to the TGF-β co-culture system (Fig. 4e). More importantly, shRNA-mediated stable silencing of IL6 in U251 cells markedly abrogated the ability of TGF-β to promote sphere formation and the expression of core cancer stemness genes at the protein level (Fig. 4f, g, h). These findings indicate that IL6 plays an import role in TGF-β-mediated promotion of GSC expansion in glioma.

### TGF-β raises IL6 secretion in tumor cells via NF-κB signaling

**Fig. 5:**
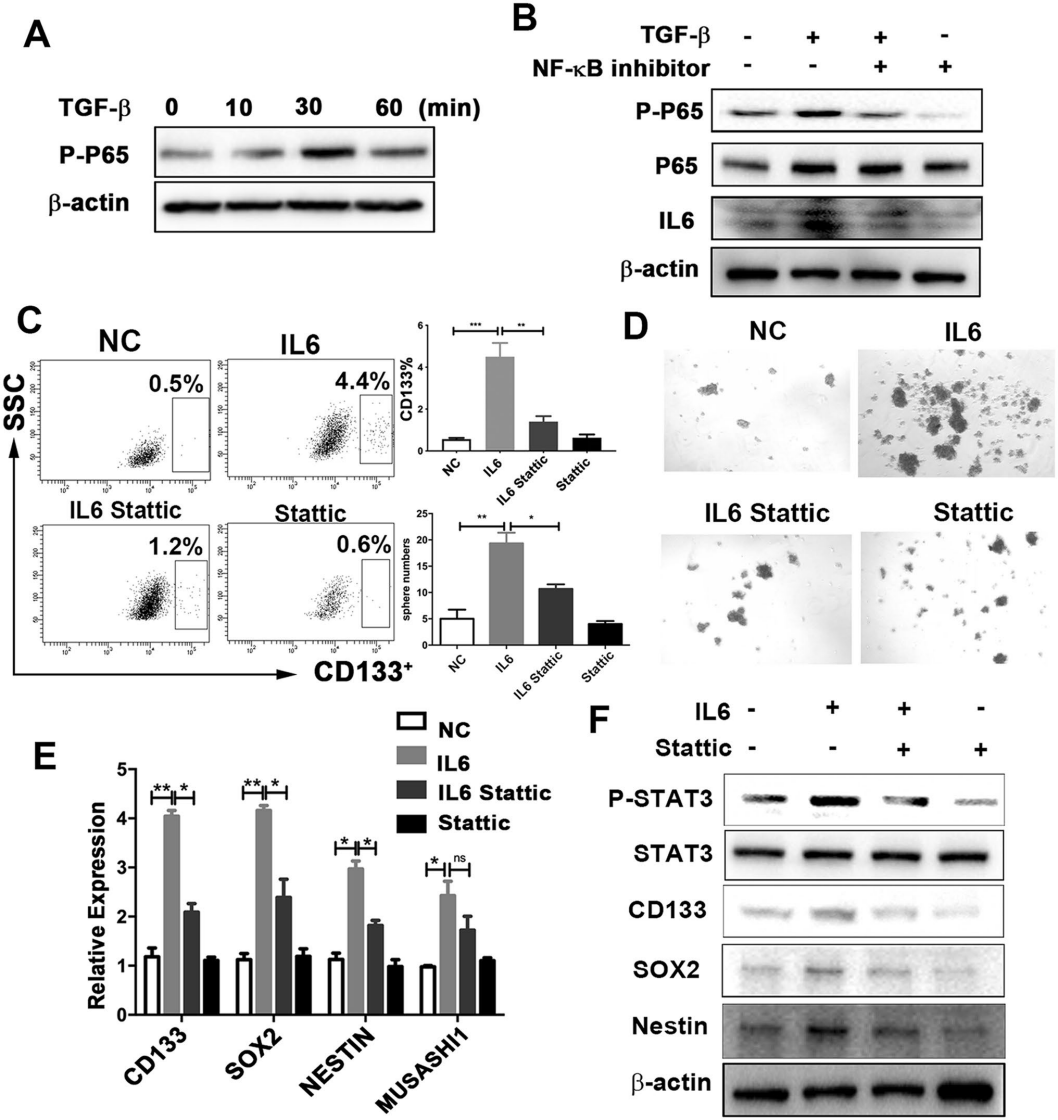
The STAT3 signaling pathway mediates IL6-induced GSC properties.** a** Expression of phospho-p65 under TGF-β treatment (10 ng/ml) at different times, assessed using western blotting analysis.** b** U251 cells were pretreated with NF-κB inhibitor (EVP4593) for 30 min, followed by TGF-β treatment (10 ng/ml) for 30 min and 24 h. The expression of phospho-p65 and IL6 was examined using western blotting analysis.** c–f**: U251 cells were pretreated with Stattic (10 umol/L, 30 min), followed by IL6 treatment (10 ng/ml, 24 h).** c** Cells were harvested to determine the proportion of CD133 cells.** d** Sphere numbers, determined using sphere-formation assay. The number of spheres per field was averaged from five random fields.** e** Expression of *CD133*, *SOX2*, *NESTIN*, and *MUSASHI*, analyzed using reverse-transcription PCR.** f** Expression of phospho-STAT3, STAT3, CD133, SOX2, and NESTIN, assessed using western blotting analysis. Results are based on three independent experiments. *P < 0.05; **P < 0.01; ***P < 0.001.* NC* normal control

Next, we examined the signaling pathway by which TGF-β increases the secretion of IL6. It has been reported that TGF-β activates IL6 expression in prostate cancer cells through the Smad or NF-κB signaling pathways [29]. Surprisingly, we found that inhibition of NF-κB, but not of Smad, abrogated expression of IL6, which induced by TGF-β (Fig. 5a, b, and Supplementary Fig. 5c). This suggests that TGF-β increases IL6 expression by activating NF-κB signaling.

### IL6 promotes glioma cancer stemness via STAT3 activation

Next, we investigated whether IL6 autocrine signaling could affect cancer cell stemness in glioma. The CD133^+^ proportion of glioma cells was elevated in the IL6 recombinant protein treatment (Fig. 5c). Consistently, IL6 promoted tumor sphere formation (Fig. 5d) and enhanced both mRNA and protein expression in stem cell genes (Fig. 5e, f). Additionally, in the clinical samples from the TCGA database, IL6 was positively correlated with CD133 expression (Supplementary Fig. 5d), which supported the in vitro results. This suggests that IL6 autocrine signaling stimulates glioma cancer stemness.

We next examined the molecular mechanisms by which IL6 promotes glioma cancer stemness. Previous studies have clearly shown that IL6 exerts its effects through the JAK1-STAT3 signal transduction pathway [30–32]. Similarly, we observed that the phosphorylation of STAT3 (p-STAT3) was activated in the IL6 recombinant protein treatment in glioma cells (Fig. 5f). Thus, we postulated that the promoting effect of IL6 on GSCs was STAT3-dependent. To this end, we utilized Stattic, a specific p-STAT3 inhibitor, to suppress the activation of STAT3 in glioma cells. In the presence of IL6, inhibition by p-STAT3 substantially reduced the proportion of CD133 cells (Fig. 5c), sphere formation (Fig. 5d), and the expression of core stemness genes at both mRNA and protein levels (Fig. 5e, f). These results indicate that STAT3 activation is necessary for IL6-promoted glioma cancer stemness.

### Tocilizumab inhibits tumor growth and cancer stemness induced by Tregs in glioma xenograft model

**Fig. 6:**
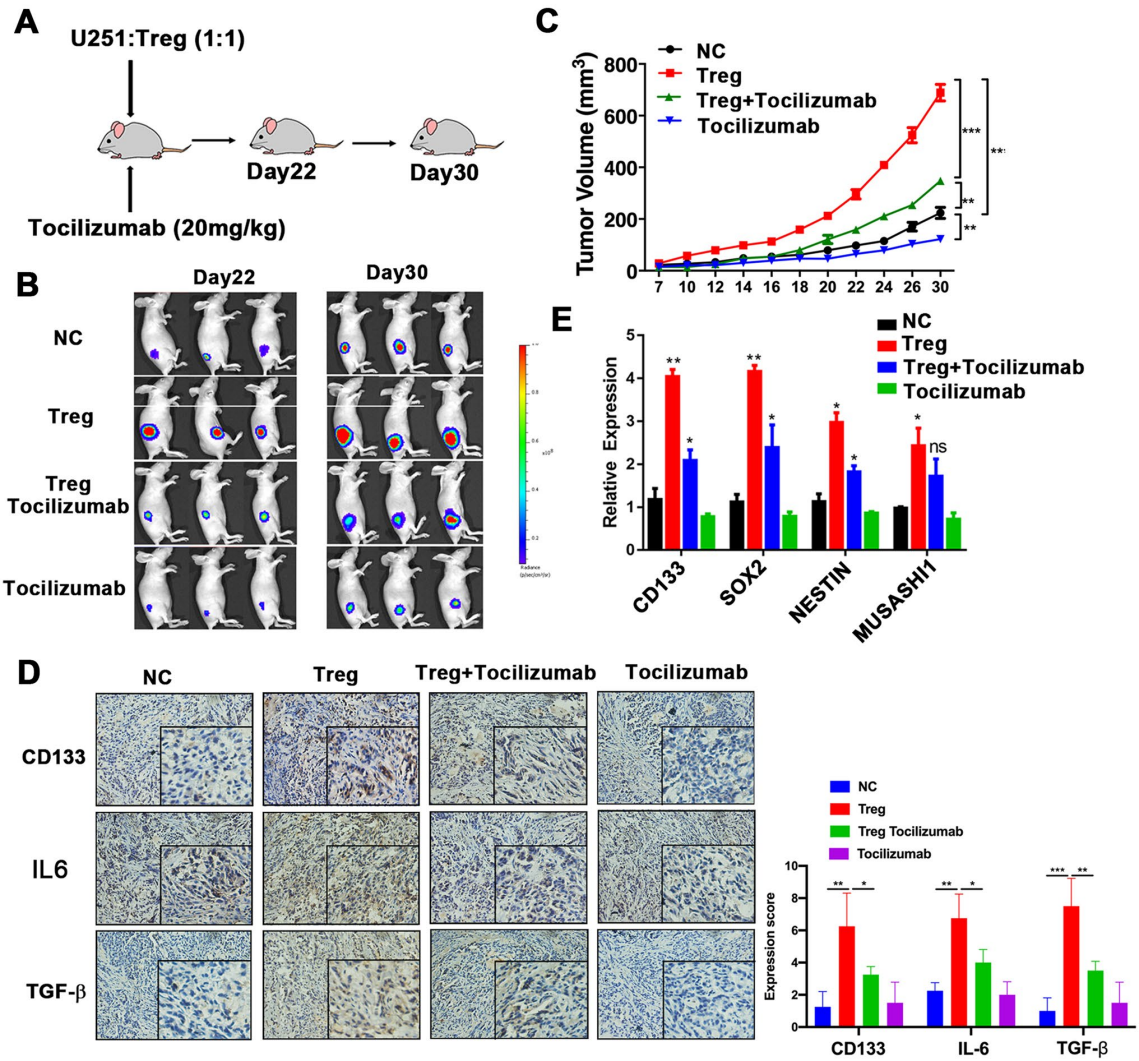
Tocilizumab blocks Tregs promotion of glioma tumor growth and stemness expansion in a mouse model in vivo.** a** In vivo experimental design.** b** Representative bioluminescent images of mouse growth, using an in vivo imaging system.** c** Tumor growth under each treatment.** d** Expression of *CD133*, *SOX2*, *NESTIN*, and *MUSASHI*, assessed using reverse-transcription PCR, in the tumor tissues of the indicated mice. *P < 0.05; **P < 0.01; ***P < 0.001

We further investigated the therapeutic potential of tocilizumab in a glioma xenograft model. Notably, injection of tocilizumab into the caudal veins of nude mice that bore a subcutaneous U251 xenograft (Fig. 6a) dramatically abolished tumor growth induced by Tregs (Fig. 6b, c). We then detected the expression of CD133, IL6, and TGF-β in the xenograft specimens using immunohistochemical staining. As expected, xenograft expression of CD133, IL6, and TGF-β in the Tregs group was significantly elevated. Combined treatment with tocilizumab substantially reduced the expression of CD133, IL6, and TGF-β induced by Tregs (Fig. 6d). To further evaluate whether treatment with tocilizumab might abrogate cancer stemness, we harvested the subcutaneous tumors for analysis of cancer stemness-associated genes at the end of the study and found that administration of tocilizumab significantly reduced the expression of cancer stemness-associated genes induced by Tregs in the xenograft specimens (Fig. 6e). Collectively, these data suggest that tocilizumab promises to be an effective therapeutic strategy in suppressing glioma cancer stemness and tumor growth.

### High expression of TGF-β–IL6–CD133 predicts poor survival in glioma patients

Finally, we further interrogated the clinical relevance of the TGF-β–IL6–CD133 signaling pathway in glioma patients. Kaplan–Meier curve analyses were performed to analyze the correlation between TGF-β–IL–6–CD133 expression and the survival of glioma patients. When we divided patients into ‘‘low’’ and ‘‘high’’ groups based on the median value of TGF-β, high TGF-β expression was associated with poor patient survival in the glioma samples and TCGA database (Supplementary Figs. 6a, 7a). Similar results were observed for IL6 expression. Overall survival was lower in patients with high IL6 expression in their glioma samples and in the glioma samples from TCGA database, than in patients with low IL6 expression (Supplementary Figs. 6b, 7b). Moreover, overall survival was lower in patients with high expression of both TGF-β and IL6 than in those with low expression of TGF-β and IL6 in their glioma samples and in the TCGA database (Supplementary Figs. 6c, 7c). These results strongly suggest that elevated expression levels of TGF-β and IL6 are significant and independent predictors of poor survival in glioma patients.

We further divided patients into ‘‘low TGF-β and low CD133′’ and ‘‘high TGF-β and high CD133’’ groups, based on expression levels in their glioma samples and glioma samples from TCGA database. Overall survival was lower in the high TGF-β and high CD133 group than in the low TGF-β and low CD133 group (Supplementary Fig. 6d, 7d). Similarly, overall survival was lower in patients with high expression of both IL6 and CD133 in their glioma samples and in the TCGA database than in other patients (Supplementary Fig. 6e, 7e). Survival was lower in patients with high expression of TGF-β, IL6, and CD133 in both the glioma samples and TCGA database than in other patients (Supplementary Figs. 6f, 7f). These results strongly suggest that the expression of genes in the TGF-β–IL6–CD133 axis could predict the prognosis for glioma patients (Supplementary Fig. 8).

## Discussion

In spite of intensive efforts and the progress achieved in tumor biology and clinical treatment of cancer, there has been little improvement in average survival for glioma patients. Thus, the failure of conventional oncologic treatment has prompted investigators to look for new and more targeted therapeutic options. The properties of cancer stem cells include the expression of stem cell markers, infinite self-renewal, sphere formation, and the capacity for multi-potential differentiation. A massive amount of evidence has shown that glioma stem cells (GSCs) can initiate highly invasive tumors [33]. GSCs have been proven to be resistant to various chemotherapeutic agents such as temozolomide, the standard chemotherapeutic agent for glioblastoma treatment, allowing these cells to survive therapy, leading to disease recurrence [34–36]. Therefore, understanding the molecular regulatory mechanisms that control GSC maintenance is central to developing novel anti-tumor therapies to achieve complete remission and tumor elimination. CSCs can actively shape their niche; conversely, the various components of the niche, including immune cells, stromal cells, cytokines, and chemokines, affect CSC properties, causing treatment to be ineffective. Therefore, it is urgent to understand the interactions between CSCs and their surrounding microenvironment. Further, investigating the molecular regulatory mechanisms that control CSC maintenance is central to developing novel anti-tumor therapies for complete remission and tumor elimination. Here, we have revealed the mechanism whereby GSC capacity is promoted by a signaling pathway in the glioma TME. Tregs promoted GSC expansion by secreting TGF-β and increasing autocrine IL6/STAT3 signaling. Moreover, these effects were blocked by tocilizumab in vivo, suggesting the potential therapeutic effect of tocilizumab on glioma patients.

As the main component of tumor-infiltrating cells in the TME, Tregs play an immunosuppressive role, enabling the tumor to escape immune-effector cell attack and immune surveillance [16, 19]. However, the mechanisms by which Treg infiltration in tumor tissues affects tumor cell function, particularly of CSCs, have not been completely elucidated. We found that Tregs promoted the proportion of CD133 in U251 cells. CD133 has been accepted as the predominant GSC marker; other GSC markers include SOX2, NESTIN, and MUSASHI1. As expected, Tregs also caused elevated expression of core stemness genes and contributed to the self-renewal capacity of GSCs. Our findings expand on the existing knowledge that Tregs promote the properties of GSCs in glioma. Our cytokine-profile screening indicated that Tregs secreted TGF-β, which promoted CSC capacity in glioma tissue. Tregs are known to be the main source of TGF-β [19]. Although it also has reported that TAMs are the source of TGF-β [37], we found that TGF-β is dominantly derived from Tregs in the TME of glioma. TGF-β is usually oncogenic in advanced tumors [38]. Specifically in high-grade glioma, the TGF-β pathway acts as an oncogenic factor. Glioma patients with high TGF-β expression had a poor prognosis [39], consistent with our findings. The link between TGF-β and CSCs has been reported for several types of cancer, including glioma. TGF-β increases the self-renewal capacity of glioma-initiating cells, via Smad-dependent induction of leukemia inhibitory factor and the subsequent activation of JAK-STAT3 signaling [40]. In addition, an autocrine TGF-β loop maintains the self-renewal capacity of glioma-initiating cells, by directly targeting the SOX2 transcription factor [39]. Our findings are consistent with these published findings. Although it has been reported that TAMs could promote GSC in different cancer types, in this report, we found another novel mechanism which Tregs also could enhance cancer stemness in glioma. TGF-β binds to type I and type II serine/threonine kinase receptors. The ligand-bound TGFBR2 is then able to efficiently trans-activate the type I TGF-β receptor (TGFBR1), which transduces intracellular signals via canonical Smad-dependent and/or Smad-independent pathways [41]. It has been reported that knockdown of TGFBR2 markedly inhibits the invasiveness of glioma stem-like cells [42]. In our study, we observed that TGFBR2 expression was higher in the glioma cell line than TGFBR1 and TGFBR3. Knockdown of TGFBR2 expression in glioma cells significantly reduced the promotion of GSC by Tregs, confirming that TGF-β-TGFBR2 mediated the role of Treg-induced GSCs properties.

Then we further investigate the remaining unknown underlying mechanisms responsible for the effects of TGF-β on GSCs. We observed that IL6 expression was dramatically higher under the TGF-β treatment, and that IL6 mediated the promotion of GSCs which was induced by TGF-β. In a different cell model, TGF-β has been reported to induce IL6 expression via a different mechanism. In human bronchial epithelial cells, TGF-β induces IL6 expression by activating Smad2 [43, 44]. In human lung fibroblast cells, TGF-β stimulates IL6 expression, and MAPK and AP-1 mediate the effect of TGF-β on IL6 expression [45]. In the present study, we observed that IL6 was activated by TGF-β in an NF-κB-dependent manner. The finding is supported by published reports that NF-κB induces IL6 expression in glioblastoma [46].

IL6, an important inflammatory cytokine, is linked to tumorigenesis, angiogenesis, and predicts poor prognosis in patients with various types of malignant tumor such as glioblastoma [47, 48]. In addition, IL6 can promote the expansion of CSCs in hepatocellular carcinoma and metastatic breast cancer [10, 49]. Targeting of IL6 signaling suppresses CSC survival and tumor growth in glioma [31, 50]. Consistent with previous reports, we found that IL6 autocrine signaling was able to enhance the characteristics of GSCs by activating STAT3. Targeting IL6 with tocilizumab significantly attenuated the expression of cancer stemness core genes in glioma. Tocilizumab is a humanized IL6 receptor antibody that competitively inhibits IL6 signaling by blocking both soluble IL6R and membrane-bound IL6R. Tocilizumab has been widely used to treat patients with rheumatoid arthritis and several chronic inflammatory diseases. In addition, various studies also demonstrated potential efficacy of tocilizumab treatment for several cancer types. So far, most reports about the mechanism that the tocilizumab inhibits the tumor growth are dependent on blocking IL6 and IL6R signaling. In our study, we found after co-culture with Treg, only tumor cell U251 produced IL6, which suggested that tocilizumab may directly act on tumor cells, not on Tregs. However, it also has been reported that tocilizumab could inhibit tumor growth through impairing tumor-related neovascularization and main proangiogenic factor VEGF-A and its transactivator HIF-1α in humanized breast orthotopic tumor xenografts [51]. Yujia Zheng also reported that tocilizumab prevented CD39 expression on NK cells induced by IL6 derived by esophageal squamous cell carcinoma cells, which suggested that tocilizumab may inhibit tumor growth via affecting NK cells [52]. In the tumor microenvironment, TAMs are mainly the source of IL6. Shanshan Wan et al. have reported that tocilizumab could sustainably decrease HCC tumor growth in vivo by inhibiting TAM-induced CSC function [10]. In addition, Yueh-ShanWeng et al. reported that IL6 could promote M2-like macrophage polarization and IL6R antibodies abrogated this effect suggesting that tocilizumab may also contribute to anti-tumor effect via TME [53]. These studies demonstrated that in addition to targeting tumor cells, tocilizumab may also influence tumor progression by affecting multiple immune cells in TME. Our in vivo findings in the glioma mouse model revealed that under tocilizumab treatment, tumor volume declined markedly, giving us more confidence about the potential therapeutic role of tocilizumab in glioma.

In this study, we have revealed the mechanism by which Tregs promotes cancer stemness in glioma, via the TGF-β–NF-κB–IL6–STAT3 signaling pathway. We highlight a new application for tocilizumab as a potential therapeutic agent that can impair the tumor progression induced by Tregs in the TME. We provide a rational basis for future clinical administration of tocilizumab in combination with TGF-β inhibitor for glioma patients.

## Supplementary Information

Below is the link to the electronic supplementary material.

Supplementary file1 (PDF 2569 kb)

## Abbreviations

CAFs: Cancer-associated fibroblasts
CNS: Central nervous system
GSCs: Glioma stem cells
MDSCs: Myeloid-derived suppressor cells
PBMCs: Peripheral blood mononuclear cells
TAMs: Tumor-associated macrophages
TCGA: The cancer genome atlas
TGF-β: Transforming growth factor-β
TILs: Tumor-infiltrating lymphocytes
TME: The tumor microenvironment
Tregs: Regulatory T cells
