# Supplementary material for: Regulatory T cells promote glioma cell stemness through TGF-β–NF-κB–IL6–STAT3 signaling
Source: Cancer Immunol Immunother. 2021 Feb 12;70(9):2601–16. doi: 10.1007/s00262-021-02872-0 (PMC8360896; doi:10.1007/s00262-021-02872-0)
Supplement: Supplementary file 1 — Supplementary file1 (PDF 2569 kb) [file 262_2021_2872_MOESM1_ESM.pdf]

Supplementary Figures

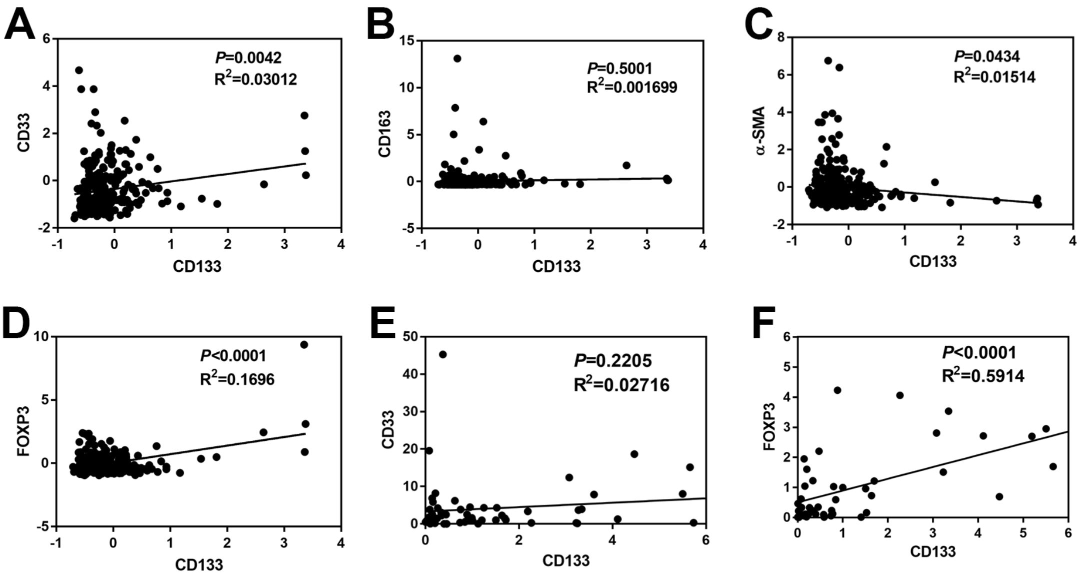

Supplementary Figure 1. Relationships between CD133 expression and various glioma markers in the TCGA database, namely (A) MDSC-marker CD33; (B) TAM-marker CD163; (C) CAF-marker α-SMA; and (D) Treg-marker Foxp3. Relationships between CD133 expression and (E) MDSC-marker CD33 and (F) Treg-marker Foxp3, in the clinical samples of glioma patients. * $P < 0.05$; ** $P < 0.01$; *** $P < 0.001$.

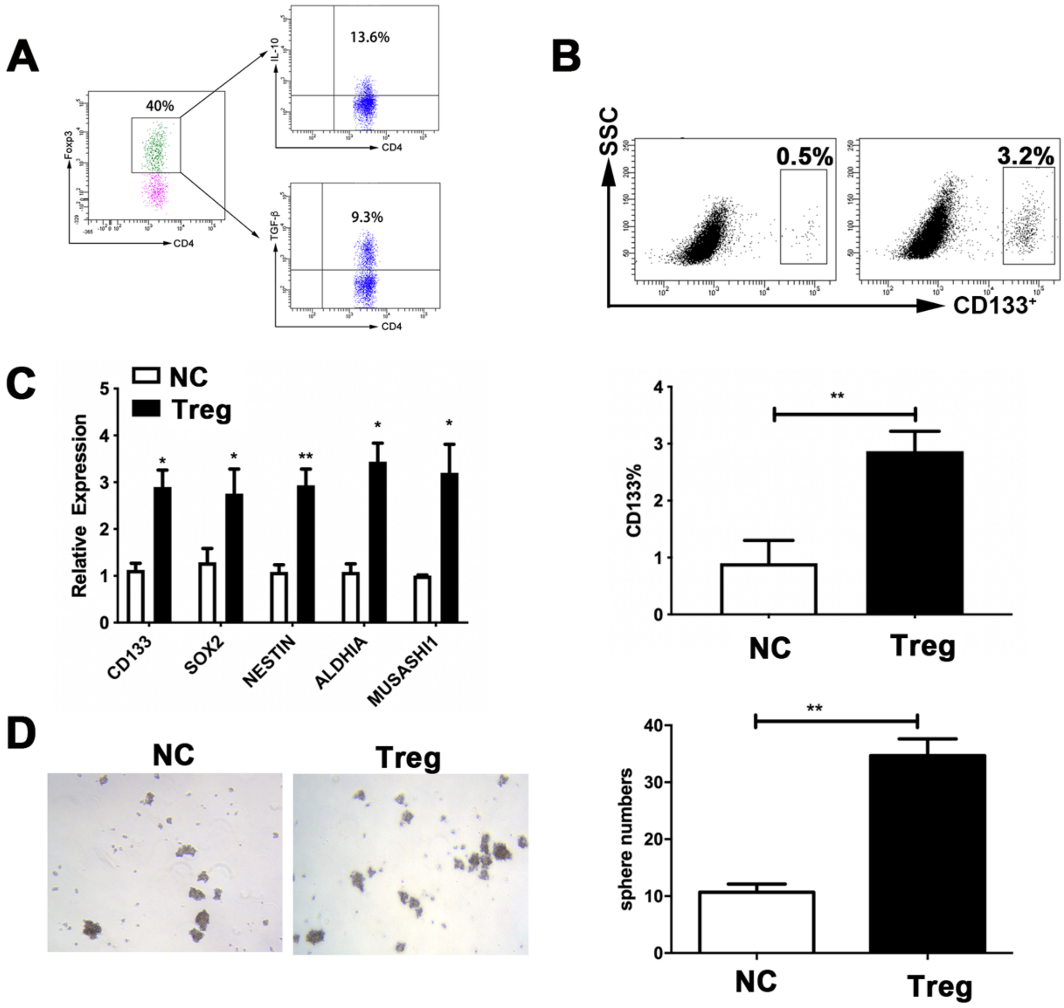

Supplementary Figure 2. (A) The function of Tregs was also validated by FACS. (B) $CD133^+$ proportions of U87 cells sorted from the peripheral blood of glioma patients, assessed by flow cytometry, following co-culture with Tregs for 24 h in Transwell chambers. (C) Expression of *CD133*, *SOX2*, *NESTIN*, *ALDH1A*, and *MUSASHI1* in the U87 cells, detected by reverse-transcription PCR after Transwell 1:1 co-culture with Tregs sorted from the peripheral blood of the glioma patients. (D) U87 cells and Tregs were co-cultured under sphere-forming conditions. The number of spheres per field was averaged from five random fields. Results are based on three independent experiments. * $P < 0.05$; ** $P < 0.01$; *** $P < 0.001$. NC: negative control.

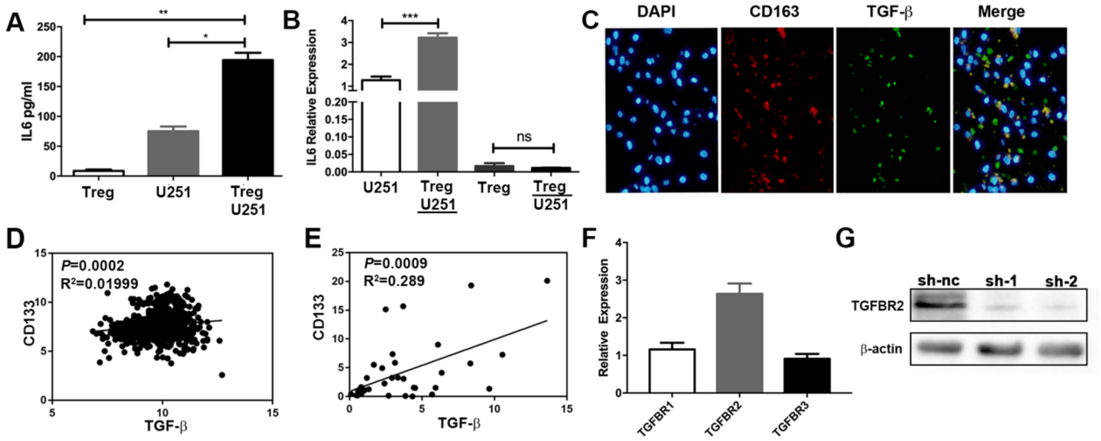

Supplementary Figure 3. (A) The IL6 protein content of cells was validated using ELISA. (B) Cells separated from the co-cultured system were evaluated for IL6 expression using reverse-transcription PCR (RT-PCR), and were compared to U251 or Tregs alone. (C) Immunofluorescence localization analysis of CD163 (red) and TGF-β (green) in glioma tissues; colocalization of CD163 and TGF-β is indicated in yellow. Scale bars: 200 μm. (D) Spearman's correlation between CD133 and TGF-β expression in 697 glioma samples from the TCGA database. (E) Spearman's correlation between CD133 and TGF-β expression in 35 clinical glioma samples. (F) TGFBR2 expression in U251 cells transfected with lentiviral sh-TGFBR2, assessed using western blotting analysis. (G) Expression of TGBFR1, TGFBR2, and TGFBR3 in U251 cells, assessed using RT-PCR. Results are based on three independent experiments. * $P < 0.05$; ** $P < 0.01$; *** $P < 0.001$.

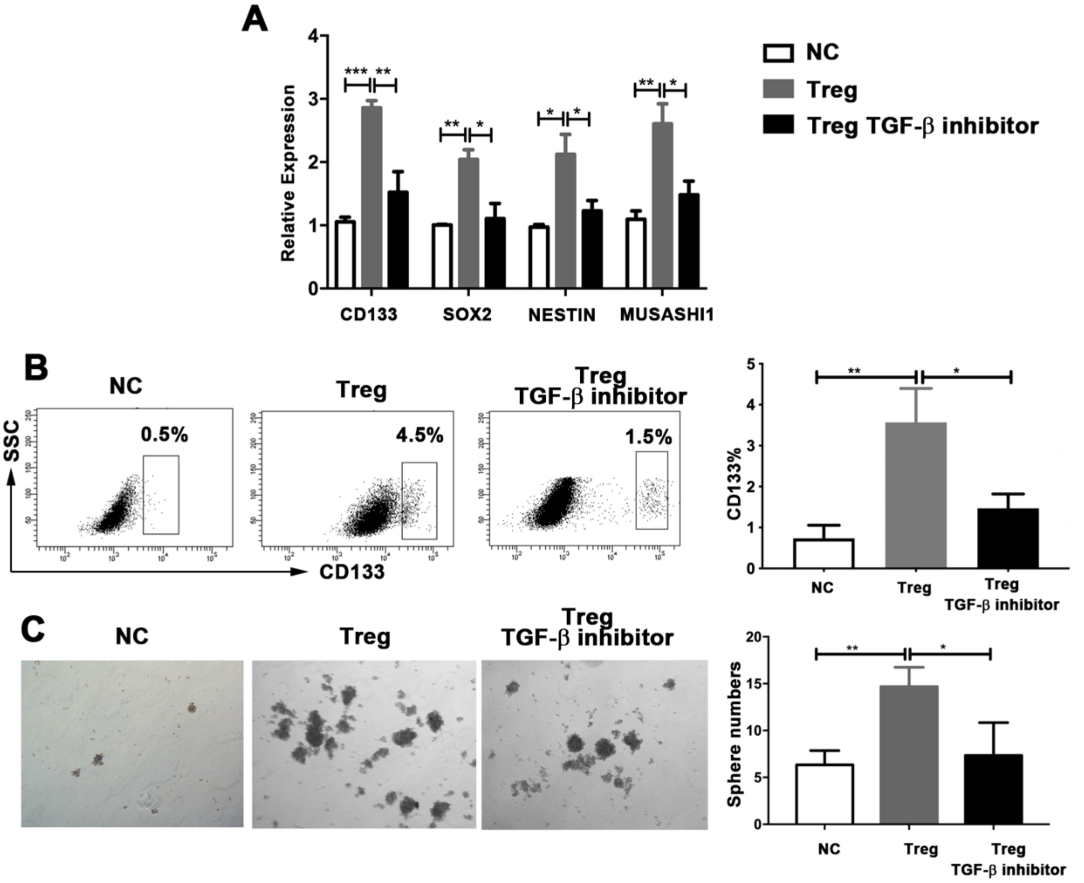

Supplementary Figure 4. (A) Expression of *CD133*, *SOX2*, *NESTIN*, and *MUSASHI1* in U251 cells, detected using RT-PCR, following cell–cell contact in a 1:1 co-culture for 24 h with Tregs sorted from the peripheral blood from the glioma patients, with or without the TGF-β-neutralizing antibody treatment (1 μg/ml). (B) Proportions of $CD133^+$ cells in the cells co-cultured with Tregs using cell–cell contact, with or without the TGF-β neutralizing antibody treatment (1 μg/ml), assessed by flow cytometry. (C) Number of spheres generated from U251 cells co-cultured with Tregs using cell–cell contact, with or without the TGF-β-neutralizing antibody (1 μg/ml) treatment. The number of spheres per field was averaged from five randomly selected fields. Results are based on three independent experiments. * $P < 0.05$; ** $P < 0.01$; *** $P < 0.001$. NC: negative control.

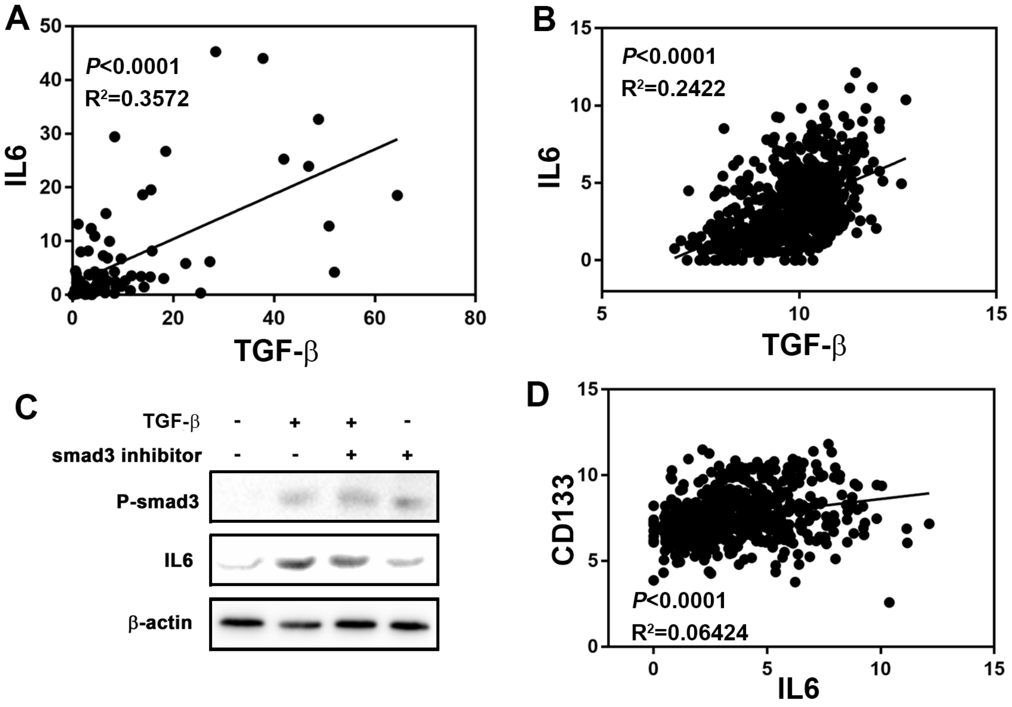

Supplementary Figure 5. (A) Spearman's correlation between TGFβ and IL6 expression in 84 clinical glioma samples. (B) Spearman's correlation between TGF-β and IL6 expression in 697 glioma samples from the TCGA. (C) U251 cells were pretreated with Smad3 inhibitor for 30 min, followed by TGF-β treatment (10 ng/ml). Expression of Smad3 and IL6 was examined by western blotting analysis. (D) Spearman's correlations between IL6 and CD133 expression in 697 glioma samples from the TCGA database. Results are based on three independent experiments. * P < 0.05; ** P < 0.01; *** P < 0.001.

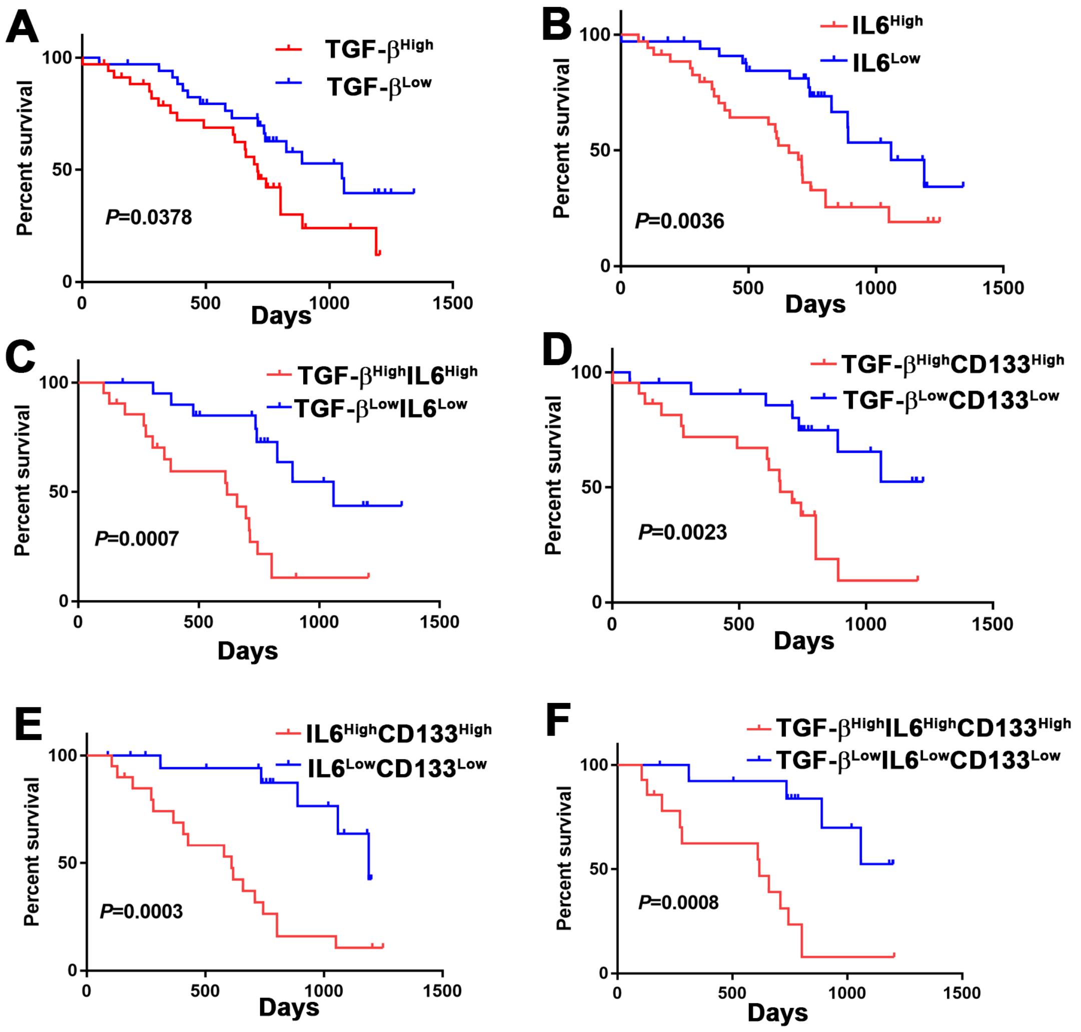

Supplementary Figure 6. Kaplan-Meier analyses of overall survival. Glioma patients with (A) low (*n*=35) and high (*n*=35) TGF-β expression; (B) low (*n*=35) and high (*n*=35) IL6 expression; (C) low (*n*=21) and high (*n*=21) TGF-β/IL6 expression; (D) low (*n*=22) and high (*n*=22) TGF-β/CD133 expression; (E) low (*n*=20) and high (*n*=20) IL6/CD133 expression; and (F) low (*n*=14) and high (*n*=14) TGFβ/IL6/CD133 expression, based on reverse-transcription PCR analysis. * $P < 0.05$; ** $P < 0.01$; *** $P < 0.001$.

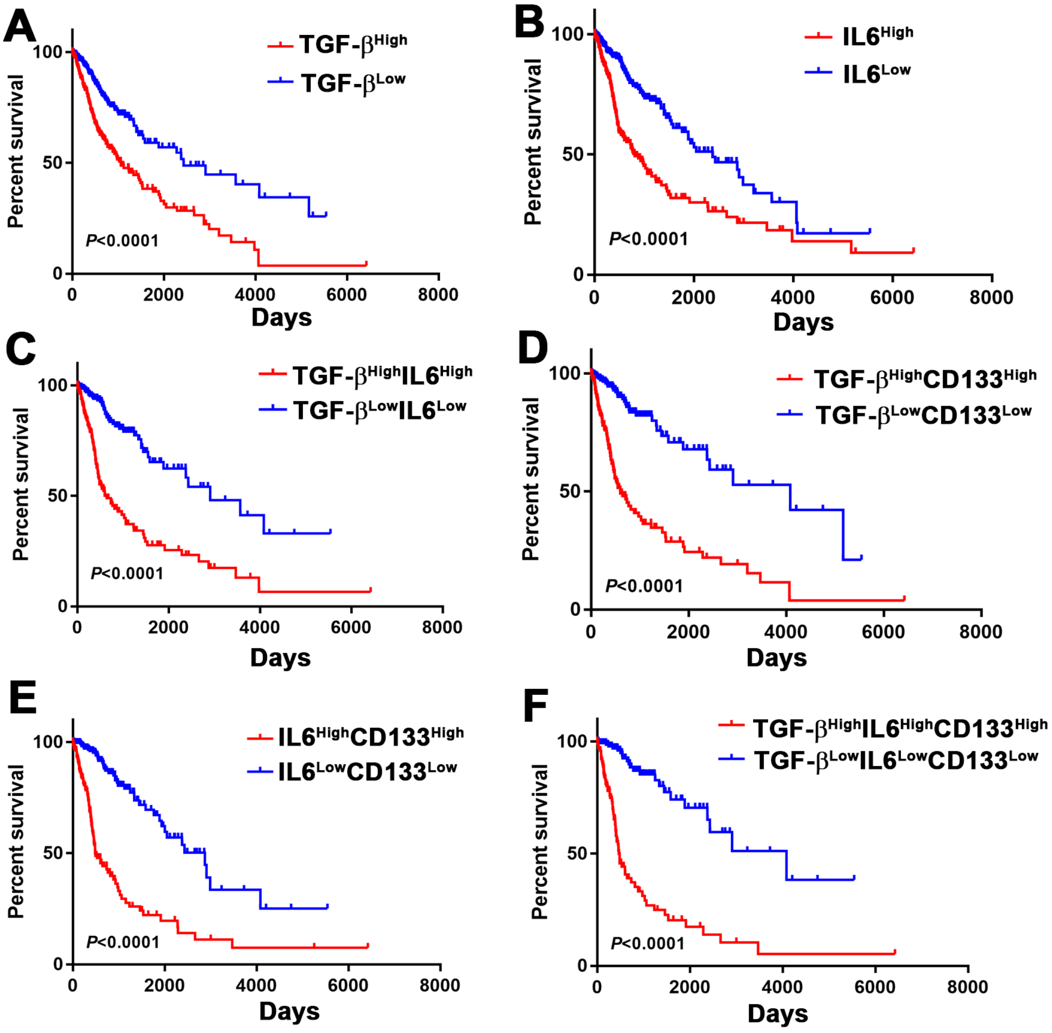

Supplementary Figure 7. Kaplan-Meier analyses of overall survival, based on data from the TCGA database. Glioma patients with (A) low (n = 317) and high (n = 381) TGF-β expression; (B) low (n = 409) and high (n = 288) IL6 expression; (C) low (n = 236) and high (n = 209) TGF-β/IL6 expression; (D) low (n = 206) and high (n = 195) TGFβ/CD133 expression; (E) low (n = 361) and high (n = 167) IL6/CD133 expression; and (F) low (n = 166) and high (n = 128) TGFβ/IL6/CD133 expression. * $P < 0.05$; ** $P < 0.01$; *** $P < 0.001$.

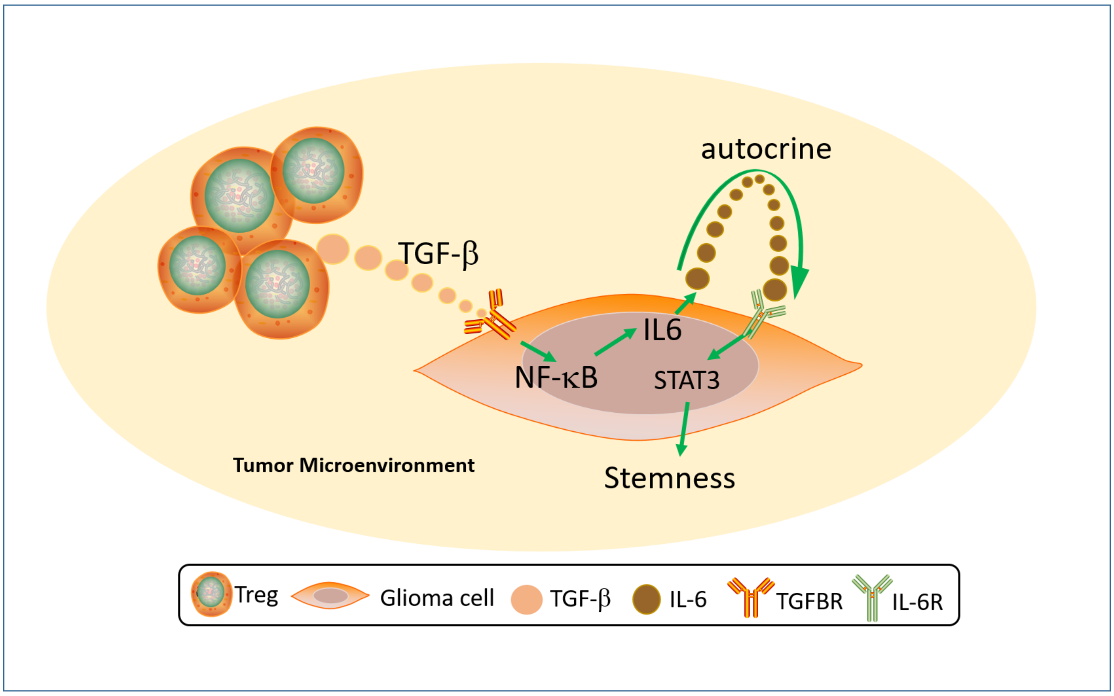

Supplementary Figure 8. Schematic diagram of the glioma tumor microenvironment described in this study. Regulatory T cells (Tregs) enhanced glioma cell stemness through the TGF-β–NF-κB–IL6–STAT3 signaling pathway.
